# Post-translational modifications in PrP expand the conformational diversity of prions *in vivo*

**DOI:** 10.1038/srep43295

**Published:** 2017-03-08

**Authors:** Patricia Aguilar-Calvo, Xiangzhu Xiao, Cyrus Bett, Hasier Eraña, Katrin Soldau, Joaquin Castilla, K. Peter R. Nilsson, Witold K. Surewicz, Christina J. Sigurdson

**Affiliations:** 1 Departments of Pathology and Medicine, UC San Diego, La Jolla, CA 92093-0612, USA; 2 Department of Physiology and Biophysics, Case Western Reserve University, Cleveland, OH 44116, USA; 3 CIC bioGUNE, Parque Tecnológico de Bizkaia, Ed. 800, Derio 48160, Spain; 4 IKERBASQUE, Basque Foundation for Science, 48013 Bilbao, Spain; 5 Department of Physics, Chemistry, and Biology, Linköping University, Linköping 581 83, Sweden; 6 Department of Pathology, Immunology, and Microbiology, UC Davis, Davis, CA 95616, USA

## Abstract

Misfolded prion protein aggregates (PrP^Sc^) show remarkable structural diversity and are associated with highly variable disease phenotypes. Similarly, other proteins, including amyloid-β, tau, α-synuclein, and serum amyloid A, misfold into distinct conformers linked to different clinical diseases through poorly understood mechanisms. Here we use mice expressing glycophosphatidylinositol (GPI)-anchorless prion protein, PrP^C^, together with hydrogen-deuterium exchange coupled with mass spectrometry (HXMS) and a battery of biochemical and biophysical tools to investigate how post-translational modifications impact the aggregated prion protein properties and disease phenotype. Four GPI-anchorless prion strains caused a nearly identical clinical and pathological disease phenotype, yet maintained their structural diversity in the anchorless state. HXMS studies revealed that GPI-anchorless PrP^Sc^ is characterized by substantially higher protection against hydrogen/deuterium exchange in the C-terminal region near the N-glycan sites, suggesting this region had become more ordered in the anchorless state. For one strain, passage of GPI-anchorless prions into wild type mice led to the emergence of a novel strain with a unique biochemical and phenotypic signature. For the new strain, histidine hydrogen-deuterium mass spectrometry revealed altered packing arrangements of β-sheets that encompass residues 139 and 186 of PrP^Sc^. These findings show how variation in post-translational modifications may explain the emergence of new protein conformations *in vivo* and also provide a basis for understanding how the misfolded protein structure impacts the disease.

Infectious prions are composed of aggregated prion protein, PrP^Sc^, and cause rapidly progressive and fatal neurodegenerative diseases^1^,^2^. Prion disorders are extraordinarily diverse, both clinically and pathologically, even among individuals expressing identical prion protein sequences. The prion protein can misfold into morphologically and biophysically distinct aggregates. Similarly, recent evidence from multiple laboratories indicates that α-synuclein, amyloid-β, and tau aggregates also exist in multiple conformations^3^,^4^,^5^,^6^,^7^, which may explain the variable clinical presentation of affected patients. In prion disease, PrP^Sc^ conformational differences are thought to be the main factor underlying the heterogeneity in the clinical phenotypes and brain lesions, even though nonproteinaceous cofactors may also contribute to this heterogeneity^8^,^9^,^10^. Distinct PrP^Sc^ conformations often co-exist, for example, two biochemically distinct subtypes of prion aggregates co-occur in the brain of approximately 39% of patients with sporadic Creutzfeldt-Jakob disease (sCJD)^11^,^12^,^13^,^14^,^15^, the most common prion disease in humans. Yet the origin of these diverse protein conformational subtypes that emerge within an individual or within a population, and how these markedly different structures impact the disease outcome, is unclear. Here we used a battery of biochemical and biophysical tools to investigate how post-translational modifications can generate new conformational variants of PrP^Sc^ *in vivo*.

The cellular prion protein, PrP^C^, consists of approximately 210 amino acids, arranged as a disordered N-terminal domain and a highly ordered C-terminal domain having three α-helices and a short, antiparallel β-sheet^16^. PrP^C^ is post-translationally modified during biosynthesis by the addition of zero to two N-linked glycans and a GPI anchor that tethers PrP^C^ to the outer leaflet of the cell membrane^17^,^18^,^19^. The number of glycans and the presence of a GPI anchor vary among the PrP molecules expressed in the brain. For example, GPI-anchorless PrP^C^ is normally shed from various cell types, including neurons and splenocytes^20^, and approximately 10% of PrP^C^ lacks the GPI-anchor in brain extracts^21^. In a hamster prion model, most brain-derived PrP^Sc^ possesses a GPI-anchor yet approximately 15% is anchorless^22^.

Certain post-translational modifications (PTMs), such as phosphorylation and deamidation, impact the fold of proteins during aggregation^23^,^24^, yet how the conformational spectrum of protein aggregates is altered by diverse post-translationally modified states is unresolved. For PrP^Sc^, accumulating evidence argues that PTMs affect the conformation. Prion-infection of knock-in mice expressing PrP with either zero, one, or two glycans led to profound strain-dependent differences in the neurons targeted by the PrP^Sc^ 25,26,27. Similarly, prion-infection of transgenic mice that express a GPI-anchorless, poorly glycosylated PrP resulted in major morphological changes in the PrP^Sc^ aggregates: GPI-anchorless PrP^Sc^ formed fibrils, whereas in wild-type (WT) mice, PrP^Sc^ lacked fibrillar morphology in the brain *in situ*^28^,^29^. However, the question remains as to whether this major difference in the morphology, and likely quaternary structure, between the PrP^Sc^ aggregates in the GPI-anchored and -anchorless states is accompanied by differences at the level of protein secondary and tertiary structure. Infrared spectroscopy studies of brain-derived GPI-anchored and -anchorless prions indicated similar global secondary structures for each of the two murine strains studied, and modest differences for a third strain^30^. Additionally, a study of GPI-anchorless prions passaged back into WT mice revealed that 1 of 2 strains maintained the original cell tropism in a cell panel assay (CPA), suggesting that some structural features can be maintained in the anchorless state^31^. We previously found that passage of one anchorless prion strain (GPI-RML, second passage) into WT mice may have led to the reemergence of the original RML strain^32^.

To better understand how PTMs impact the conformation of protein aggregates and how PTMs underlie the generation of new strains *in vivo*, here we investigate the fidelity of diverse prions during transit into a GPI-anchorless state and then back into a GPI-anchored state. Previous studies showed that GPI-anchorless RML and 22 L were similar clinically, histopathologically, and biochemically in the anchorless state after 1–3 passages, however, it was unclear whether the two anchorless strains had converged into the same strain^32^. Here we compared four serially passaged mouse-adapted prion strains (RML, 22 L, ME7, and mCWD^33^,^34^,^35^) in transgenic mice expressing GPI-anchorless PrP [Tg(GPI-PrP) mice]. The four strains in the anchorless state converged into similar appearing fibrillar prions histologically, and the mice showed equivalent incubation periods and angiocentric plaques in the brain, however, clear biochemical differences in PrP^Sc^ were identified, indicating that structural variation in the original PrP^Sc^ aggregates persisted in the GPI-anchorless prions. To then determine whether passage of the GPI-anchorless prion strains into WT mice would lead to the convergence into a single strain, formation of new strains, or reversion to the original strains, we passaged the anchorless prions back into mice expressing GPI-anchored PrP and compared the original and post-GPI-adapted strains using a battery of histological, biochemical, and biophysical assays. We found that the conformational fidelity of two strains was precisely maintained and readily recovered in WT mice, a third strain showed modest differences as compared to the original strain, and the fourth strain was profoundly modified by passage through the anchorless state as noted by HXMS, lesion profiles, and biochemical assays, resulting in the generation of a new prion strain *in vivo*.

## Results

### Diverse prion strains in GPI-anchorless mice show a convergence of clinical and histological features

**Figure 1:**
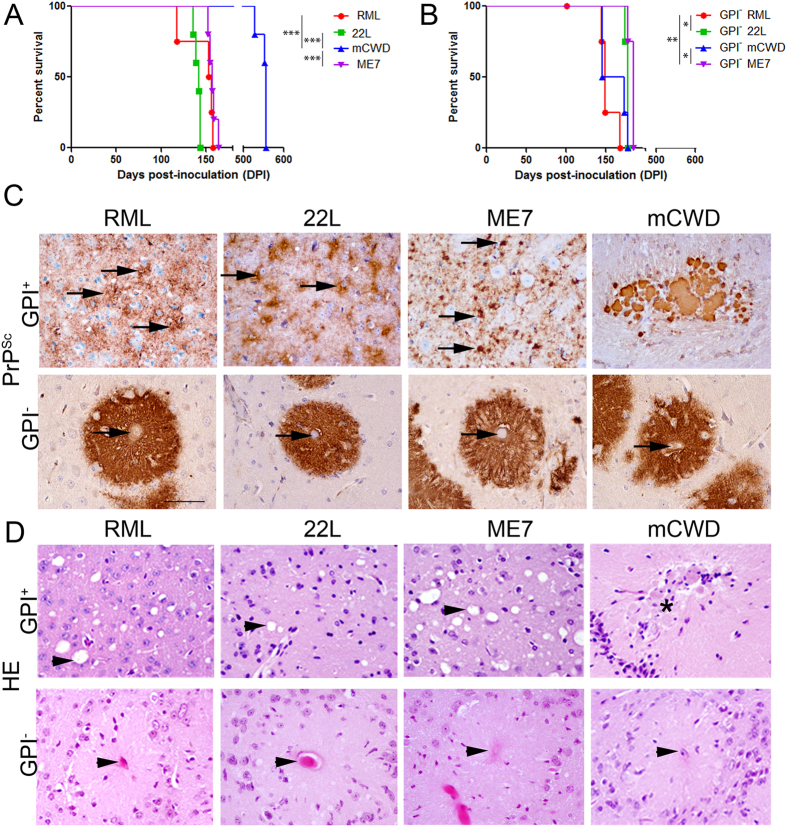
Tg(GPI-PrP) mice infected with four prion strains show similar incubation periods and pathology in brain. (**A**,**B**) Survival curves of WT (**A**) and Tg(GPI-PrP) (**B**) mice inoculated with RML, 22 L, mCWD and ME7 prion strains show variable incubation periods in the WT mice, but similar incubation periods in the Tg(GPI-PrP) mice after 4–5 passages. (**C**) PrP^Sc^ aggregates in the brain of prion-infected WT or Tg(GPI-PrP) mice. In WT mice (GPI^+^), RML forms diffuse, patchy aggregates (arrows), 22 L forms fine aggregates on cell membranes (arrows), ME7 forms dense, punctate aggregates (arrows), and mCWD forms large, dense plaques. Corresponding anchorless prions (GPI^−^) all form dense plaques that infiltrate and extend beyond a central vessel (arrows) and are present in all brain regions. Brain regions shown: RML: thalamus; 22 L: dorsal striatum; ME7: dorsal striatum; mCWD: corpus callosum; GPI-anchorless strains: cerebral cortex. (**D**) Prion-infected WT mice show spongiform degeneration in the brain after infection with 3 of 4 strains (arrow heads). mCWD plaques (asterisk) lead to very little spongiform change. Note the central vessel and lack of spongiform degeneration in prion-infected Tg(GPI-PrP) mice (arrow head). Scale bar = 100 μm. N = 4–5 mice/group. One-way ANOVA followed by Tukey’s multiple comparison test for survival times, *P < 0.05; **P < 0.05; ***P < 0.001.

The incubation period, clinical disease, and brain regions targeted (lesion profile) vary widely among mice infected with different mouse-adapted prion strains. To determine how the disease phenotype changes upon adaptation to a GPI-anchorless state, we serially-passaged RML, 22 L, ME7 and mCWD prions in Tg(GPI-PrP) mice, and refer to the resulting GPI-anchorless prions as GPI^–^ RML, GPI^−^ 22 L, GPI^−^ mCWD, and GPI^−^ ME7. RML, 22 L, and ME7 are biologically cloned prion strains originating from sheep scrapie^36^, whereas mouse-adapted mCWD originates from chronic wasting disease prions in mule deer^33^. The incubation period of the original strains in WT mice ranged from 141 to 550 days post-inoculation (dpi)] and was consistent within a strain, but varied significantly among the strains (Fig. 1A) (mean 254 ± 42 dpi; P < 0.0001, ANOVA). The first 1–3 passages were previously reported for GPI^−^ RML, GPI^−^ 22 L, and GPI^−^ mCWD^32^. However, here we found after 4–5 serial passages of all four strains in the GPI-anchorless mice, the incubation period converged to approximately 168 ± 4 dpi, although significant differences between the incubation periods for most groups were noted (Fig. 1B) (GPI^−^ RML: 152 ± 5 dpi; GPI^−^ 22 L: 176 ± 1 dpi; GPI^−^ ME7: 182 ± 2 dpi; GPI^−^ mCWD: 160 ± 7 dpi; for four strain comparison, *p* < 0.005, ANOVA).

Prion strains show a striking diversity in the PrP^Sc^ aggregate morphology and distribution throughout the brain. We and others previously found that RML, 22 L, and ME7 infection of WT mice resulted in fine to punctate PrP^Sc^ aggregates deposited diffusely in different brain regions (Fig. 1C), whereas mCWD formed large dense plaques in the corpus callosum, meningeal vessels, and periventricular regions. In the GPI-anchorless state, all four strains converged to similar large dense plaques within and surrounding blood vessels (Fig. 1C) and ventricles in most brain regions, and did not change over 4–5 passages. Plaques showed subtle differences in having sharp or indistinct plaque borders, depending on the strain. All four GPI-anchorless vasotropic strains showed plaques with little to no spongiform degeneration (Fig. 1D).

**Figure 2:**
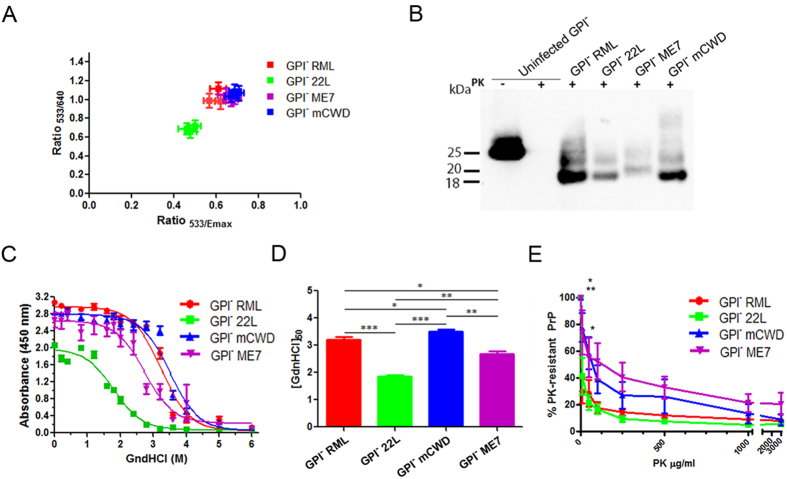
Biochemical properties of the GPI-anchorless prions. (**A**) PTAA labelling of GPI-anchorless prions. GPI^−^ 22 L shows significant differences in the ratios of emitted light intensity at ratios of 533 nm/640 nm and 533 nm/emission maximum compared with the 3 other anchorless prion strains. (**B**) Electrophoretic mobility of GPI^−^ RML, ^−^22 L, ^−^mCWD and ^−^ME7 prions reveals that the GPI^−^ ME7 PrP^Sc^ has a higher molecular weight than the other three strains, indicative of a longer PK-resistant PrP^Sc^ core fragment. (**C**) GPI^−^ RML, ^−^22 L, ^−^mCWD and ^−^ME7 exposed to different concentrations of GdnHCl prior to PK digestion show significant differences in the aggregate stability among the strains. Plotted are the mean and standard error (SE) of the remaining PrP^Sc^ as measured by ELISA from 3–4 mice per strain, each run in triplicate. (**D**) The [GdnHCl_1/2_] values for each strain reveal significant differences between the strains (one-way ANOVA followed by Tukey’s multiple comparison test). (**E**) The resistance of GPI-anchorless PrP^Sc^ to proteinase K shows a significant differences between the strains. (two-way ANOVA followed by Bonferroni post-tests of GPI- anchorless prions revealed significant differences in GPI^−^ RML vs GPI^−^ mCWD at 10 μg/ml PK**, GPI^−^ RML vs GPI^−^ ME7 at 10 μg/ml PK*, and GPI^−^ 22 L vs GPI^−^ mCWD at 10 and 50 μg/ml PK*). *P < 0.05; **P < 0.01; ***P < 0.001.

Although all four prion strains induced disease following similar incubation periods and showed similar angiocentric plaques in the GPI-anchorless mice, the fibril structure may differ among the strains. Luminescent conjugated polymers (LCP) are amyloid-binding molecules used to distinguish prion strains, as the emission spectra varies depending on the structure of the amyloid bound^37^. We compared the emission spectra of the LCP, polythiophene acetic acid (PTAA), bound to prions in brain sections. After three serial passages, GPI^−^ RML, GPI^−^ ME7, and GPI^−^ mCWD prion plaques showed a similar green-shifted PTAA emission spectra that differed significantly from that of GPI^−^ 22 L, suggesting that certain anchorless PrP fibrils were structurally distinct (Fig. 2A).

### Strain-specific differences in the biochemical properties of GPI-anchorless PrP^Sc^

To determine whether GPI-anchorless PrP^Sc^ differs biochemically, we compared the resistance to proteinase K (PK) cleavage, electrophoretic mobility, and conformational stability among the four anchorless prion strains. PK cleaves the relatively unstructured N-terminal part of PrP^Sc^, resulting in a PK-resistant PrP^Sc^ core that varies with the PrP^Sc^ conformation^38^. We found that the PK-resistant PrP^Sc^ core size was similar for GPI^−^ RML, GPI^−^ 22 L, and GPI^−^ mCWD prions, yet was longer for the GPI^−^ ME7 prions (Fig. 2B). This is consistent with previous HXMS studies, which revealed that the PK-resistant core of PrP^Sc^ from GPI^−^ RML and GPI^−^ 22 L prions starts at residue 81, whereas that of GPI-ME7 prions has an N-terminus starting at residue ~73/77^39^.

In previous studies, GPI-RML PrP^Sc^ and GPI^–^ 22 L PrP^Sc^ were more stable in chaotropes than their respective anchored counterparts^32^. Here the conformational stability of the 3^rd^ passage of GPI-anchorless strains was assessed by denaturing aliquots of PrP^Sc^ with 0 to 6 M guanidine hydrochloride (GdnHCl), digesting with PK, quantifying the PrP^Sc^, and calculating the [GdnHCl]_1/2_, which is the GdnHCl concentration at which half the PrP^Sc^ remains^40^. Consistent with previous measurements^32^, we found that the absence of a GPI-anchor resulted in the formation of highly stable prions (Fig. S1A), with [GdnHCl]_1/2_ values ranging from 1.87 (GPI^−^ 22 L) to 3.51 (GPI^−^ mCWD), and strains were significantly different from each other (Fig. 2C and D).

The GPI-anchorless strains were also compared for their resistance to increasing concentrations of PK and, similar to the stability, GPI-anchorless prions varied from each other (Fig. 2E) and were generally more resistant to PK digestion than their GPI-anchored counterparts (Figs S1B and S2). Among the GPI-anchorless strains, GPI^−^ mCWD and GPI^−^ ME7 PrP^Sc^ were more resistant to PK digestion than GPI^−^ RML or GPI^−^ 22 L prions (Fig. 2E). Thus, although the GPI-anchorless prions led to similar incubation periods and lesion distribution in the brain of infected mice, the differences in the LCP emission spectra, PK cleavage sites, conformational stability, and PK degradation profile collectively indicate that the GPI-anchorless fibrils remain as distinct conformational variants of PrP^Sc^.

### C-terminus of GPI-anchorless PrP^Sc^ is less accessible to H/D exchange following multiple passages

For all four strains, the incubation period progressively shortened with passage in the GPI-anchorless mice, although the plaque-bound PTAA emission spectra was unchanged (Fig. S3). Previously, GPI^−^ RML showed a gradual decrease in the conformational stability with each subsequent passage in the Tg(GPI-PrP) mice, suggesting that the prion conformation changed during three serial passages^32^. To test this possibility and gain insight into the nature of these conformational changes, we used two complementary methods: backbone amide hydrogen/deuterium exchange coupled with mass spectrometry (HXMS) and histidine hydrogen/deuterium exchange mass spectrometry (His-HXMS). These mass spectrometry based methods are one of very few tools available for structural analysis of brain-derived protein aggregates such as PrP^Sc^ 39,41.

**Figure 3:**
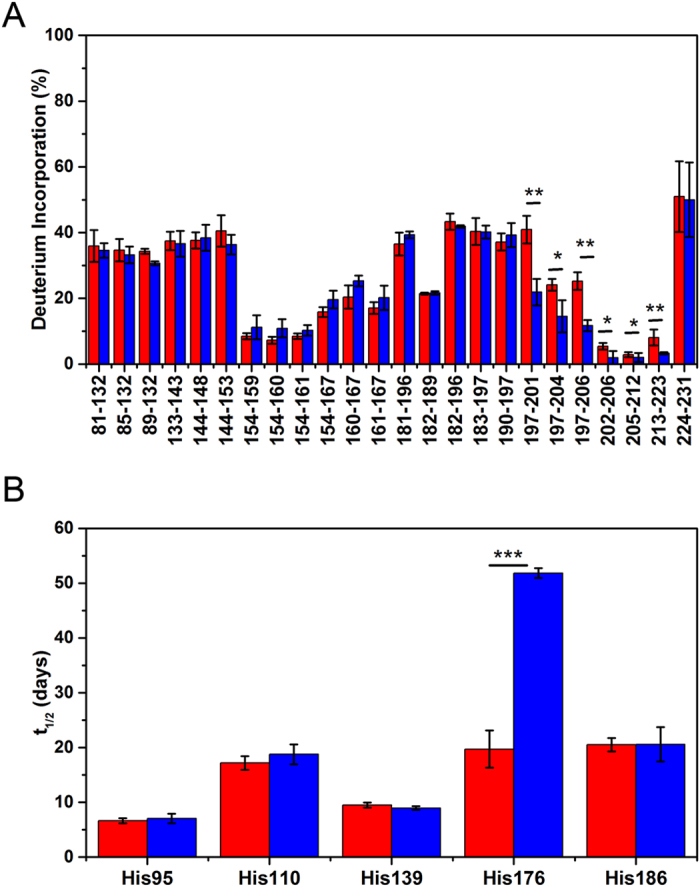
Structural comparison of GPI-anchorless RML prions at different passages by mass spectrometry based methods. (**A**) Backbone amide H/D exchange for PrP^Sc^ purified from GPI-anchorless PrP mice after the first (red) and fourth (blue) passage of RML prions. The samples were incubated in D_2_O for 24 hours at 37 °C, and deuterium incorporation for each peptic fragment derived from these samples was assessed by mass spectrometry. (**B**) Histidine H/D exchange for first (red) and fourth (blue) passage PrP^Sc^. The parameter t_1/2_ represents the half-time of exchange reaction for individual His residues. Error bars indicate standard deviation based on three experiments. Student’s t-test: *P < 0.05; **P < 0.01; ***P < 0.001.

In the HXMS method, one monitors the rate of H/D exchange of backbone amide hydrogens. While this exchange is very fast within the unstructured protein regions, it becomes much slower for systematically hydrogen bonded β-strands that are building blocks of amyloids^42^ and constitute the proteinase-resistant core of PrP^Sc^ 39,41,43,44,45,46,47. In HXMS, the exchange rates are assessed for peptic fragments that can be separated by liquid chromatography and identified by MS, providing segment-specific structural information. Figure 3A shows the extent of deuterium incorporation after 24 hours of incubation in D_2_O for PrP^Sc^ derived from Tg(GPI-PrP) mice after the first and fourth passages of RML prions. The degree of exchange for both passages appears to be essentially identical for all peptic fragments derived from the PrP^Sc^ region up to residue ~196. However, substantial passage-dependent differences could be detected within the C-terminal segment between residues ~197 and 223, with the fourth passage GPI^−^ RML PrP^Sc^ consistently showing higher protection against H/D exchange compared to the first passage prions. As discussed previously^39^,^41^, these C-terminal region-specific differences may reflect factors such as different proportions of residues involved in β-strands and turns between them and/or packing differences between individual β-strands.

The nature of structural differences between PrP^Sc^ from the first and fourth passage of RML prions in mice expressing GPI-anchorless PrP was further probed using His-HXMS. This relatively novel approach monitors the rate of H/D exchange for C2 protons in histidine imidazole rings^48^,^49^. In contrast to backbone amide HXMS that probes structural organization at the level of the polypeptide backbone, His-HXMS reports on the microenvironment (water accessibility) of individual His side chains. The latter method proved very useful in studies of amyloids and related protein aggregates, providing information about packing arrangement and interfaces between β-sheets^39^,^41^,^50^. In particular, recently His-HXMS allowed detection of region-specific packing differences between two major strains of CJD prions^41^.

There are five His residues in the PK-resistant region of mouse PrP^Sc^. As shown in Figure 3B, four of them (His95, His110, His139 and His186) are characterized by a very similar environment in the first and fourth passage GPI^−^ RML PrP^Sc^, as indicated by essentially identical exchange half-times. In contrast, pronounced differences are observed for His176. The latter side chain is substantially more protected from water in fourth passage GPI^−^ RML PrP^Sc^ compared to first passage GPI^−^ RML PrP^Sc^, suggesting distinct packing arrangements and/or interfaces between β-sheets around residue 176.

### Defining the strain recovered after passage through the GPI-anchorless prion state

**Figure 4:**
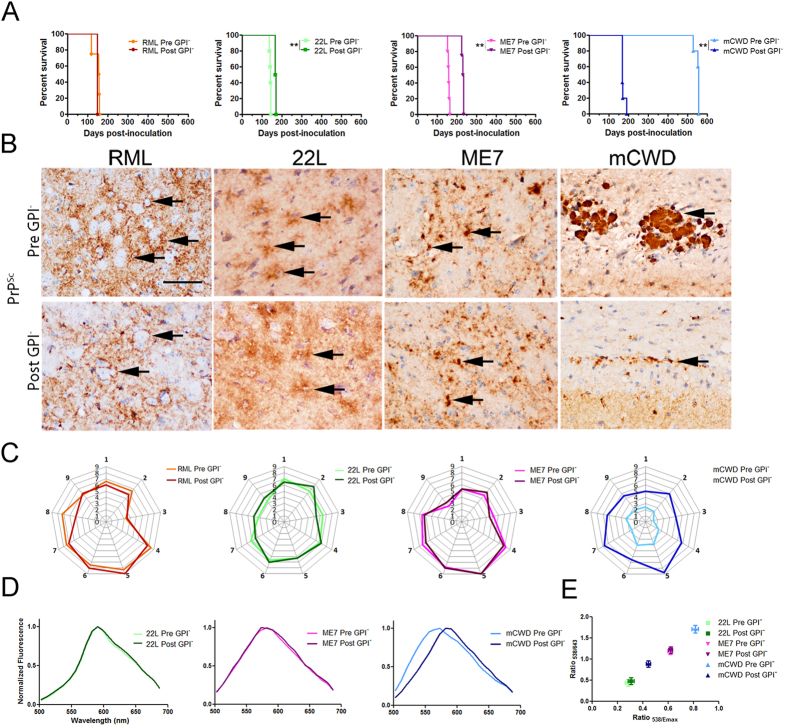
mCWD prions form a new prion strain following passage through the GPI-anchorless mice. (**A**) Survival curves of WT mice inoculated with RML, 22 L, ME7 and mCWD (Pre GPI^−^) and GPI-anchorless RML, 22 L, ME7 and mCWD (Post GPI^−^) prions. mCWD shows a significantly shortened survival time after passage through the anchorless state, whereas ME7 prions show a modest lengthening in the survival time. (**B**) Morphology of PrP^Sc^ aggregates in the brains of prion-infected WT mice inoculated with the original strains and GPI-anchorless prions. GPI^−^ RML, ^−^22 L, ^−^mCWD and ^−^ME7 passaged into WT mice show diffuse to punctate PrP^Sc^ aggregates (arrows) similar to those induced by the original RML, 22 L, and ME7 strains. In contrast, GPI-mCWD led to small clusters of plaques in WT mice, differing markedly from the large, discrete dense plaques of original GPI-anchored mCWD, although both plaque types were enriched in the corpus callosum (arrows). (**C**) Lesion profile of WT mice infected with Pre and Post GPI^−^ prion strains. For the Pre and Post GPI^−^ RML, ^−^22 L and ^−^ME7 infected WT mice, the severity of spongiosis, astrogliosis, and PrP^Sc^ deposition were scored for nine brain regions (see Methods) and were nearly superimposable. For Post GPI^-^ mCWD, the regions and severity of spongiosis, astrogliosis, and PrP^Sc^ distribution were more widespread and severe as compared to Pre GPI^−^ mCWD. Radial plots show the mean from 4–7 mice per strain. (**D**) Emission spectra of PTAA-labelled Pre and Post GPI^−^ prions in the brain of WT mice (for 22 L and ME7) and in the brain of *tg*a20 mice (for mCWD prions) measured at wavelengths from 500–700 nm. (**E**) Ratios at 538 nm/643 nm and at 538 nm/emission maximum are shown. Brain regions shown in (**B**): RML, Pre: thalamus, Post: dorsal striatum; 22 L, Pre and Post: cerebral cortex; ME7, Pre and Post: dorsal striatum; mCWD, Pre and Post: corpus callosum. N = 4–5 mice per group. Scale bar = 100 μm. Logrank test for survival times, *P < 0.05; **P < 0.01; ***P < 0.001.

To determine whether PrP^Sc^ conformation of the original strain may be retained in the GPI-anchorless fibrils, we inoculated all four anchorless prions into WT mice. We compared the incubation period, aggregate morphology and lesion profile with those induced by the original prion strain. Passage of GPI^−^ RML prions into WT mice led to an incubation period that was similar to that observed with the original RML prions, at 146 ± 3 days versus 147 ± 0 days, respectively, whereas GPI^−^ 22 L and GPI^−^ ME7 prions led to a modestly prolonged incubation period in WT mice. In contrast, GPI^−^ mCWD prions in WT mice showed a dramatic 3-fold reduction in the incubation period when compared to the original strain in WT mice (Fig. 4A and Table 1).

Transmission barriers in prion disease are suggested by a progressive decrease in the incubation period upon serial passage. To determine whether the incubation period would decrease with serial passage, we performed two serial passages of the GPI-anchorless prions in *tg*a20 mice, which overexpress WT PrP by approximately 4–6 fold^51^. GPI^−^ RML and GPI^−^ mCWD prions in *tg*a20 mice showed only subtle differences in the incubation period between the first and second passages suggestive of a stable strain, whereas GPI^−^ 22 L and GPI^−^ ME7 prions showed a decrease in the incubation period upon second passage (Fig. S4).

Despite the slight change in incubation period for the 22 L and ME7 prions following passage through the anchorless state, the PrP^Sc^ aggregate morphology and lesion profiles were identical to the original strains. (Fig. 4A). WT mice inoculated with GPI^−^ 22 L and GPI^−^ ME7 prions showed fine to coarse PrP^Sc^ aggregates (Fig. 4B) with spongiform change and astrogliosis predominating in regions typical of the original 22 L and ME7 (Fig. 4C). Similarly, GPI^−^ RML prions in WT mice showed diffuse aggregates similar to the original RML strain with slight differences in the severity of gliosis and PrP^Sc^ deposition in the cerebellar peduncle (Fig. 4B and C). Remarkably, the pathology in WT mice inoculated with GPI^−^ mCWD prions had markedly changed, as plaques were widespread and appeared smaller and less distinctly defined, clearly differing from the sharply demarcated, dense congophilic plaques typical of mCWD (Fig. 4B). Similar histological findings were also observed in *tg*a20 mice inoculated with GPI^−^ mCWD (Fig. S5A), further supporting that a novel mCWD prion strain arose or was selected following passage through the GPI-anchorless state. The lesion profile of the mCWD strain also changed between the first and second passage, with increased PrP^Sc^ deposition and gliosis on the second passage in nearly every brain region (Fig. S5A and B). However, there were no changes in the electrophoretic mobility of the PrP^Sc^ between the 1^st^ and 2^nd^ passage of GPI^−^ mCWD (Fig. S5C).

The PTAA emission spectra for the WT mice infected with GPI^−^ 22 L or GPI^−^ ME7 prions were nearly indistinguishable from that of the original 22 L and ME7 strains (Fig. 4D and E), while no PTAA labelling of the GPI^−^ RML in WT mice was detected, as previously observed for RML^32^. In contrast, the GPI^−^ mCWD in *tg*a20 mice showed a significantly red-shifted emission spectra of PTAA bound to PrP^Sc^ as compared to that of the original mCWD strain in *tg*a20 mice (Fig. 4D and E).

### Certain prions differ biochemically after passage through the GPI-anchorless state

**Figure 5:**
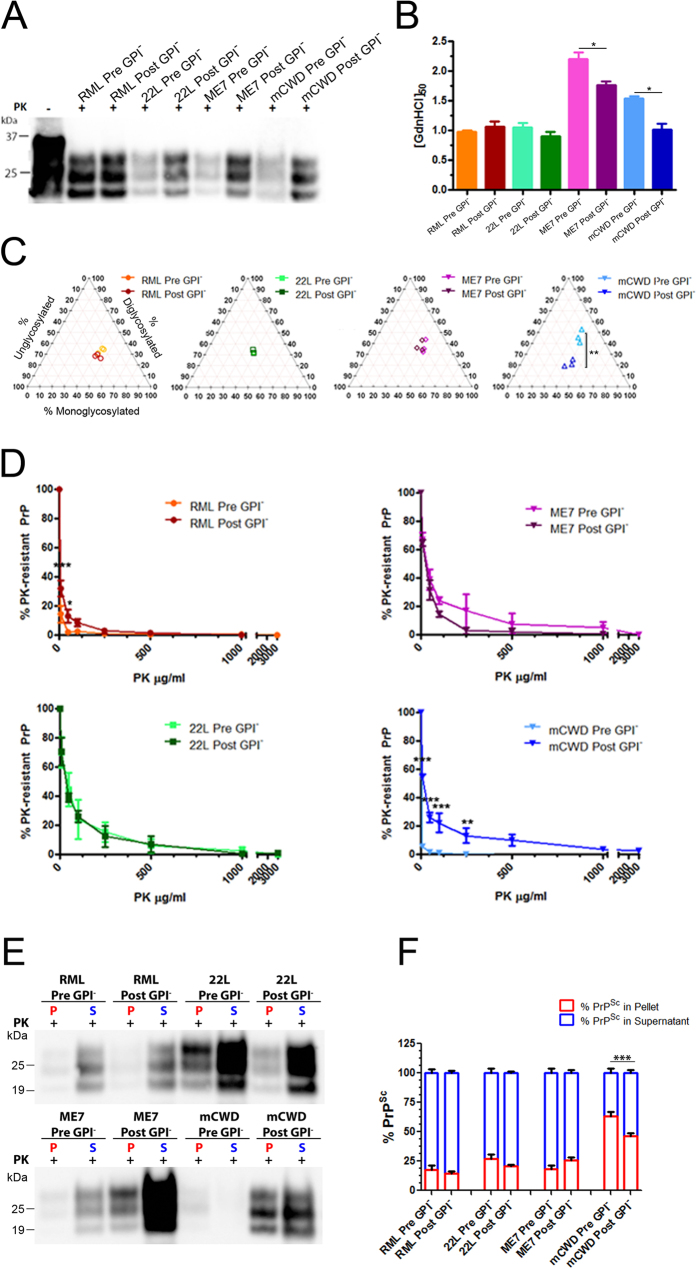
mCWD prions post-GPI-passage differ biochemically from the original mCWD strain. (**A**) The Post GPI^−^ RML, ^−^22 L, ^−^ME7 and ^−^mCWD strains show no differences in electrophoretic mobility as compared to the original strains. (**B**) [GdnHCl_1/2_] of Pre and Post GPI^−^ strains show that RML and 22 L have not changed following passage through an anchorless state whereas ME7 and mCWD prions are less stable than the original strain. (**C**) The di-, mono-, and unglycosylated PrP bands of PK-digested Pre and Post GPI^−^ RML, ^−^22 L, ^−^ME7 and ^−^mCWD strains strains were measured following western blotting. Triplots show similar glycoform profiles for all strains except mCWD, which varied significantly from the original strain (2-tailed, unpaired Student’s *t* test). (**D**) PK-resistance of Post GPI^−^ -RML and −22L were similar to their respective Pre GPI^−^ strains, while Post GPI-ME7 show lower PK resistance than -ME7 and Post GPI-mCWD prions show higher PK resistance than–mCWD. Two-way ANOVA followed by Bonferroni post-tests of Pre and Post GPI^−^ prions revealed significant differences in RML at 10 and 50 μg/ml PK, and mCWD at 10, 50, and 100 μg/ml and at 250 μg/ml PK. (**E**) Western blots show the solubility of Pre and Post GPI^−^ RML, ^−^22 L, ^−^ME7 and ^−^mCWD strains. S: supernatant and P: pellet. (**F**) Quantification of the pellet fraction for all strains (3 animals per strain). Two-way ANOVA followed by Bonferroni post-tests of Pre and Post GPI^−^ prions revealed significant differences in the PrP^Sc^ solubility of Pre- and Post GPI-mCWD prions. *P < 0.05; **P < 0.01; ***P < 0.001.

To assess the biochemical properties of the GPI-anchorless prions in WT mice, the electrophoretic mobility, conformational stability, glycoprofile, and PK resistance of PrP^Sc^ were measured. The electrophoretic mobility of the GPI^−^ RML, GPI^−^ 22 L and GPI^−^ ME7 PrP^Sc^ in WT mice resembled that observed for original anchored prions (Fig. 5A). The conformational stability of the GPI^−^ RML and GPI^−^ 22 L PrP^Sc^ in WT mice revealed a low resistance to GdnHCl denaturation, similar to that of the original anchored RML and 22 L PrP^Sc^ ([GdnHCl]_1/2_ values of pre- and post- GPI^−^ RML: 0.98 versus 1.07; pre- and post- GPI^−^ 22 L: 1.05 versus 0.90) (Fig. 5B). In contrast, GPI^−^ ME7 and GPI^−^ mCWD in WT mice yielded prions that were less stable than the original ME7 and mCWD (ME7: pre-GPI^−^: 2.20 versus post-GPI^−^: 1.77; mCWD: pre-GPI^−^: 1.54 versus post-GPI^−^: 1.01) (Fig. 5B). Intriguingly, the glycoprofile of GPI^−^ mCWD PrP^Sc^ in WT mice differed from the original mCWD PrP^Sc^ in WT mice in showing a relative increase in the mono- and unglycosylated bands (Fig. 5C).

Consistent with the stability and glycoprofile results for three strains, the resistance to PK degradation also reverted back to the low PK resistance observed for the original WT strains, RML, 22 L, and ME7 (Pre GPI^−^ versus Post GPI^−^, at 10 μg/ml PK, *P* < 0.001; at 50 μg/ml PK, *P* < 0.05; Two-way ANOVA followed by Bonferroni post-tests) (Fig. 5D and S2B). The mCWD strain became more resistant to PK degradation after passage through the anchorless state, again suggesting that the mCWD prions had either formed a new structure, or that an mCWD substrain was selected by GPI-anchorless PrP^C^ (Pre GPI^−^ versus Post GPI^−^ mCWD at 10, 50, and 100 μg/ml PK: *P* < 0.001; at 250 μg/ml PK, *P* < 0.01; Two-way ANOVA followed by Bonferroni post-tests) (Fig. 5D). Finally, we measured the ratio of soluble to insoluble PrP^Sc^ for each strain before and after passage through the GPI-anchorless state. Although there were no differences in the ratio of soluble to insoluble PrP^Sc^ for RML, 22 L, or ME7 strains, mCWD prions became significantly more soluble following passage through the GPI-anchorless state (Fig. 5E and F).

### His-HXMS analysis of GPI-anchored prion strains

**Figure 6:**
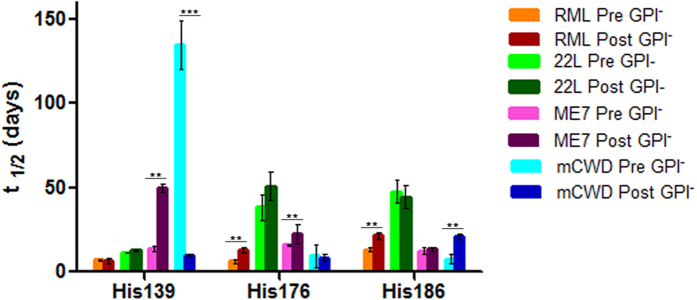
H/D exchange for GPI-anchored prion RML, 22 L, ME7 and mCWD before and after passage through GPI-anchorless mice. Data for His95 and His110 was incomplete due to low abundance of peptide fragments containing these residues and is not shown. The parameter t_1/2_ represents the half-time of exchange reaction for individual His residues. Error bars indicate standard deviation based on three experiments. **P < 0.01; ***P < 0.005.

The biochemical assays suggest that the PrP^Sc^ aggregate structure of at least some prion strains could be altered by pre-passage through mice expressing GPI-anchorless PrP. To further explore this issue, we performed His-HXMS measurements on PrP^Sc^ from the original (GPI-anchored) prions and the same strains after passage through the GPI-anchorless state. These experiments revealed that, for most strains studied, this pre-passage has a significant effect on the environment of His side chains in PrP^Sc^ aggregates (Fig. 6), indicating differences in PrP^Sc^ structure at the level of packing arrangements and interfaces between β-sheets (see discussion of the His-HXMS method above). These differences are particularly pronounced around His139 for mCWD prions and, to a somewhat lesser degree, ME7. Structural differences are also detectable by His-HXMS between pre- and post- GPI^−^ RML PrP^Sc^, even though they appear to be more modest and localized to a more C-terminal part of the protein (i.e., around His176 and His186). In contrast, no such differences could be detected for PrP^Sc^ associated with 22 L prions.

## Discussion

Protein aggregates, including those composed of tau, amyloid-β, and α-synuclein can form conformationally distinct structures^11^,^12^,^13^,^14^,^15^, yet the factors contributing to this conformational diversity are poorly understood. The prion protein also misfolds into a wide range of conformations, often with multiple forms co-occurring within an individual^6^,^7^,^8^,^9^,^10^. Here we show that four prion strains serially passaged in GPI-anchorless mice led to fibrillar, angiocentric prions associated with a nearly identical clinical and pathological disease phenotype. However, these four fibrillar strains differed biochemically, suggesting that the secondary and tertiary structure varied within the fibrils, in agreement with IR studies^30^.

On first passage into the GPI-anchorless mice, all four strains switched to a new pathological phenotype, with prions arranged in large angiocentric plaques throughout the brain, as previously reported^28^,^32^. Anchorless plaques are composed of fibrils^29^, suggesting that the GPI-anchor obstructs fibril assembly in WT mice. Each subsequent prion passage in the GPI-anchorless mice led to a decrease in the incubation period and conformational stability of PrP^Sc^, although the plaques appeared unchanged histologically and by PTAA emission spectral analysis. HXMS experiments revealed a higher degree of protection against H/D exchange in the C-terminal region of PrP^Sc^ near the N-glycan sites (N180, N196) after multiple passages, suggesting that the C-terminal region had become more ordered with passage. Furthermore, differences were observed in the packing of the β-sheets around His176 (i.e., in the vicinity of one of the glycosylation sites). Thus, the poorly glycosylated state of the GPI-anchorless prions may enable a more ordered packing arrangement in the distal C-terminus.

To determine whether the PrP^Sc^ structure of the original strain was maintained within the fibril, GPI-anchorless prions were passaged back to WT mice. Two strains, RML and 22 L, showed little or no change following passage through an anchorless state. The incubation period, pathology, biochemical profile and certain biophysical characteristics of PrP^Sc^ such as conformational stability were nearly identical to those in the corresponding original strains and were similar to an earlier passage of GPI-RML into WT mice^32^, even though the His-HXMS data suggest modest packing alteration for RML PrP^Sc^ after passage through the anchorless state. These observations, together with the finding that global secondary structures of anchored and anchorless RML and 22 L prions are similar^30^, suggest that these anchorless prions maintain structural features of the original RML and 22 L strains, even though they assemble into fibrils. The morphological differences between the GPI-anchored and anchorless PrP^Sc^ (subfibrils versus fibrils) are likely related to distinct packing arrangements of β-sheets.

The post GPI^−^ ME7 prion strain subtly differed from the original strain, but these differences were less dramatic compared to those observed for mCWD. The incubation period had become prolonged and the PrP^Sc^ stability was lower than for the original ME7, suggesting that the structure had changed. This structural alteration of PrP^Sc^ was further confirmed by His-HXMS data that indicate distinct packing arrangements, especially around His139. In this context, it should be noted that an elegant report by Baron and colleagues using infrared (IR) spectroscopy on highly purified anchored and anchorless ME7 PrP^Sc^ noted subtle differences in the secondary structure of ME7 following conversion into GPI-anchorless ME7, indicating that the anchorless state may alter the structure of certain prion strains^30^. The structure of GPI-anchored ME7 prions emerging upon passage of GPI-anchorless ME7 in WT mice was not examined by IR spectroscopy.

Mahal and colleagues also performed two serial passages of RML and ME7 in GPI-anchorless mice and found that passage of anchorless RML into WT mice led to a novel strain, based on Cell Panel Assay (CPA) studies of cell tropism^31^. In the present study, differences in the aggregate morphology or lesion profile in mice infected with GPI^-^RML were not detected, however there were subtle differences in the His-HXMS of the aggregates that may translate into the differences reported with the CPA. Both studies were in agreement in that the incubation period, western blotting, and conformational stability assay were unchanged in anchorless RML-infected WT mice. The CPA comparison of the original and Post-GPI passaged ME7 strain showed no differences in the strain, whereas we report subtle differences in the biological readout (incubation period) and in the conformational stability of the prions, as well as differences in the His-HXMS of pre- and post-GPI passaged ME7 around His139.

Post-GPI- mCWD prions led to the emergence of a new strain in WT mice by all measurements. GPI^−^mCWD-infected WT mice showed marked alterations in the biochemical and biophysical properties of PrP^Sc^ and profound changes in the incubation period, clinical disease, and pathology. Certain aspects of the plaque morphology and brain deposition pattern observed for the new mCWD strain were reminiscent of the original strain (for example, plaque deposition in the corpus callosum), suggesting that some structural features of the original mCWD PrP^Sc^ might have been retained. Biochemical studies indicated that the Post GPI^−^ mCWD PrP^Sc^ was more soluble and PK resistant, and less stable in chaotropes, and had an altered glycosylation profile and PTAA emission spectra. Additionally, the microenvironments (water accessibility) of histidine side chains at position 139 and, to a lesser degree 186, were markedly different in the original and post GPI^−^ mCWD PrP^Sc^ (Fig. 6), indicating altered packing arrangements of β-sheets in the vicinity of residues 139 and 186. Thus, PrP^Sc^ generated by the GPI^−^ mCWD prions in WT mice assembled into a structure distinct from the original mCWD, indicating that the variation in PTMs either generated or selected (from substrains) a new, rapidly replicating strain.

GPI-anchorless mice express poorly glycosylated PrP. Altering PrP glycans modifies the prion conformation in knock-in mice expressing zero, one, or two glycans^25^, yet interestingly, none of the mice expressing specific glycans formed strictly angiocentric plaques as observed in the GPI-anchorless mice, suggesting this vascular tropism is due to the lack of a membrane anchor. Considering that approximately 10–15% of PrP^C^ is released from the cell membrane as GPI-anchorless PrP^21^, and prions may be switching between the anchored and anchorless state *in vivo*, these results suggest that GPI-anchorless PrP^C^ molecules within the brain can expand the conformational spectrum of prions and serve as a source for new prion strains within an individual and within a population. Low levels of glycans on PrP may also play a role in the generation of new strains^27^, yet how conversion of poorly glycosylated PrP modifies the PrP^Sc^ structure is not clear.

Certain anchored and anchorless prions (RML and 22 L) may maintain the same global secondary structure within non-fibrillar aggregates or fibrils, however the disease that ensues profoundly differs by nearly every measure, including clinical signs, organ tropism, neuroinvasive ability, plaque distribution, and biochemical properties of PrP^Sc^ (Table 2). As compared to the anchored prions, anchorless RML and 22 L prions (i) accumulate in many additional tissues, including kidneys and adipose tissue^28^,^32^,^52^,^53^, (ii) form extensive angiocentric plaques distributed throughout the brain^28^, (iii) cause a longer clinical disease phase^29^, and (iv) no longer readily spread into the CNS from extraneural sites^32^,^54^. Anchorless prions also accumulated to high titers in the blood and caused an amyloid-induced cardiomyopathy as well as cerebral amyloid angiopathy in the Tg(GPI-PrP) mice^52^. These findings suggest that the prion disease phenotype may be largely determined by the PrP^Sc^ oligomerization state (fibrillar lar or nonfibrillar morphology) that, in turn, is likely dependent on packing arrangements between β-sheets.

Post-translational modifications commonly occur in protein biosynthesis and include phosphorylation, lipidation, glycosylation, amidation of the C-terminus, acetylation, and disulfide bond formation. Deamidation of asparagine and glutamine side chains is a common, nonenzymatic, spontaneous change that can lead to major changes in protein stability and alterations in fibril structure^23^. For prions, the role of N-linked glycans in defining strain characteristics for some strains has been demonstrated^25^,^27^,^55^. GPI anchors are present on approximately 250 proteins and have also been extensively shown to impact protein structure. For example, removing the GPI^−^ anchor from diverse proteins by phosphatidylinositol-specific phospholipase C (PIPLC) significantly alters their enzymatic activity^56^,^57^,^58^,^59^. By identifying the effect of the PTMs on the disease outcome for four prion strains, we have revealed PTMs as a source of novel conformations of ordered protein aggregates *in vivo*. This finding has implications for other PTMs that may also have a similar effect on the conformation of a protein aggregate *in vivo*. In a broader context, one could argue that the post-translationally modified state of the protein may contribute to the remarkable diversity of protein aggregate structures derived from the same primary amino acid sequence in patients with various neurodegenerative diseases.

In addition to providing evidence for how new strains may arise *in vivo*, the use of Tg(GPI-PrP) mice has proven useful in establishing the relationship between the PTMs and the assembly pathway of prion protein aggregates. Four prion strains cause a nearly identical disease in GPI^−^anchorless mice, yet retain structural variations within the fibrils and features of the original strain. Simply removing the GPI^−^ anchor and most glycans from PrP^C^ enabled fibril assembly to occur, and passage into WT mice led to apparent recovery of the original non-fibrillar prions for the RML and 22 L strains. Finally, the fibrillar GPI-anchorless RML and 22 L prions caused similar diseases in the Tg(GPI-PrP) mice, yet differed profoundly from the subfibrillar RML and 22 L prions in WT mice. This finding suggests that the PrP^Sc^ oligomerization state (fibrillar versus non-fibrillar morphology) may be one of the important determinants governing the disease incubation period, organ tropism, and aggregate spread to the brain. Further studies are needed to determine the relationship between the oligomerization state and specific structural features such as the tertiary structure of individual monomers, their packing arrangement, and the interfaces between β-sheets.

## Materials and Methods

### Ethics statement

All procedures involving animals were performed to minimize suffering and were approved by the Institutional Animal Care and Use Committee at UC San Diego (IACUC number: S08037). Protocols were performed in strict accordance with good animal practices, as described in the *Guide for the Use and Care of Laboratory Animals* published by the National Institutes of Health.

### Prion inoculations

Tg(GPI-PrP) mice were line 44 mice homozygous for the GPI- transgene backcrossed to Prnp null mice (C57BL/6 background)^28^. All prion strains have been maintained in C57BL/6 mice, with the exception of mCWD, which was maintained in *tg*a20 mice^33^. mCWD was propagated in *tg*a20 mice, and the 5^th^ passage was used. Homogenates from individual animals were used for the inoculation. WT (C57BL/6), *tg*a20, or Tg(GPI^−^ PrP) mice (groups of n = 4–6 mice) were anesthetized with ketamine and xylazine and intracerebrally inoculated into the left parietal cortex with 30 μl of prion-infected brain homogenate prepared from terminally ill mice. Mice were monitored three times weekly, and prion disease was diagnosed according to clinical criteria including ataxia, kyphosis, stiff tail, hind leg clasp, and hind leg paresis. The incubation period was calculated from the day of inoculation to the day of terminal clinical disease. Mice were euthanized at the onset of terminal disease. The brain was halved, and one hemi-brain was formalin-fixed, then immersed in 96–98% formic acid for 1 hour, washed in water, and post-fixed in formalin for 2–4 days. Brains were then cut into 2 mm transverse sections and paraffin-embedded for histological analysis. The remaining hemi-brain was cut and a 2–3 mm transverse section at the level of the hippocampus/thalamus was embedded in OCT and immediately frozen on dry ice. The remaining brain sections were frozen for biochemical analyses. Mice were maintained under specific pathogen-free conditions.

### Histopathology and immunohistochemical stains

Four μm sections of brain were cut onto positively charged silanized glass slides and stained with hematoxylin and eosin, or immunostained using antibodies for PrP (SAF84) or GFAP for astrocytes. For PrP staining, sections were deparaffinized and incubated for 5 min in 96% formic acid, then washed in water for 5 min, treated with 5 μg/ml of proteinase-K for 7 min, and washed in water for 5 min. Sections were then placed in citrate buffer (pH 6) and heated in a pressure cooker for 20 min, cooled for 5 min, and washed in distilled water. Sections were blocked and incubated with anti-PrP SAF-84 (SPI bio; 1:400) for 45 min followed by anti-mouse biotin (Jackson Immunolabs; 1:250) for 30 min, followed by streptavidin-HRP (Jackson Immunolabs; 1:2000) for 30 min. Slides were then incubated with DAB reagent for 7 min and an enhancer for 2 min (Invitrogen). Sections were counterstained with hematoxylin. GFAP immunohistochemistry for astrocytes (1:500; DAKO) was similarly performed, however with antigen retrieval by PK-digestion (20 μg/ml for 10 min at room temperature).

### Lesion profile

We selected 9 anatomic brain regions in accordance with previous strain-typing protocols from 4–5 mice per group^36^,^60^. We scored spongiosis, gliosis, and PrP immunological reactivity on a scale of 0–3 (not detectable, mild, moderate, severe). A sum of the three scores resulted in the value obtained for the lesion profile for the individual animal and was depicted in the ‘radar plots’. Numbers correspond to the following brain regions: (1) dorsal medulla, (2) cerebellum, (3) hypothalamus, (4) medial thalamus, (5) hippocampus, (6) septum, (7) medial cerebral cortex dorsal to hippocampus, (8) cerebral peduncle, (9) cerebellar peduncle. Two investigators blinded to animal identification performed the histological analyses.

### Western blotting and sodium phosphotungstic acid precipitation

Brain tissue was homogenized in PBS using a Beadbeater™ tissue homogenizer. Homogenates in a Tris-based lysis buffer (10 mM Tris-HCl, 150 mM NaCl, 10 mM EDTA, 0.5% NP40, 0.5% DOC; pH 7.4) were digested with 50 μg/ml proteinase K at 37 °C for 30 min and the reaction stopped by boiling samples for 5 min in LDS loading buffer (Invitrogen). Samples were electrophoresed in 10% Bis-Tris gel (Invitrogen) and transferred to a nitrocellulose membrane by wet blotting. Membranes were incubated with monoclonal antibody POM19 (discontinuous epitope at C-terminal domain, amino acids 201–225^61^, a kind gift from Dr. Adriano Aguzzi) followed by incubation with an HRP-conjugated anti-mouse IgG secondary antibody. The blots were developed using a chemiluminescent substrate (ECL detection kit, ThermoScientific) and visualized on a Fuji LAS 4000 imager. Quantification of PrP^Sc^ glycoforms was performed using Multigauge V3 software (Fujifilm).

PrP^Sc^ was concentrated from *tg*a20 mouse brain samples by performing sodium phosphotungstic acid (NaPTA) precipitation prior to western-blotting^62^. Briefly, 100 μl aliquots of 10% brain homogenate in an equal volume of 4% sarkosyl in PBS were digested with an endonuclease [BenzonaseR (Sigma)] followed by treatment with 20 μg/ml proteinase K at 37 °C for 30 min. After addition of NaPTA, MgCl_2_, and protease inhibitors (Complete-TM, Roche), extracts were incubated at 37 °C for 30 min, and centrifuged at 18,000 g for 30 min at 37 °C. Pellets were resuspended in 0.1% sarkosyl prior to electrophoresis and blotting.

### Conformation stability assay

Prion strain stability in GdnHCl was performed as previously described^40^. Briefly, lysed brain homogenates were denatured in GdnHCl ranging from 0–6 M for 1 hr. Samples were then diluted with a Tris-based lysis buffer (10 mM Tris-HCl, 150 mM NaCl, 10 mM EDTA, 4% sarkosyl; pH 7.5) to 0.15 M GdnHCl and digested with PK at a ratio of 1:500 (1 μg PK: 500 μg total protein) for 1 hr at 37 °C. Digestion was stopped with 2 mM phenylmethylsulfonyl fluoride (PMSF) and Complete TM protease inhibitor (Roche) followed by centrifugation at 18,000 g for 1 hr. Pellets were washed in 0.1 M NaHCO_3_ (pH 9.8) and centrifuged at 18,000 g for 20 min. Pellets were then denatured in 6 M guanidine isothiocyanate (GdnSCN), diluted with 0.1 M NaHCO_3_, and coated passively onto an ELISA plate. PrP was detected with biotinylated-POM1 antibody (epitope in the globular domain, amino acids 121–231^61^), a streptavidin HRP-conjugated secondary antibody, and a chemiluminescent substrate. Each strain (n = 3–5 mice per strain) was analyzed in at least 3 separate experiments. Statistical analysis was performed using a Student’s t test.

### Proteinase K (PK) resistance

Brain homogenate was diluted in Tris-based lysis buffer (50 mM TrisHCl, 137 mM NaCl, and 2% sarkosyl; pH 8.0), mixed for 15 min at 25 °C at 1000 rpm, and divided into 8 tubes. Tris lysis buffer containing 2% sarkosyl was added to each tube and samples were digested with increasing concentrations of PK (10 to 3,000 μg/ml). Samples were then incubated for 2 hr at 37 °C at 1000 rpm and the PK digestion stopped by boiling for 5 min in LDS loading buffer (Invitrogen) prior to western blot and development using POM19. PrP signals from each PK digested sample was captured and quantified using a Fujifilm LAS-4000 imager and Multigage software. Each strain was analyzed in at least 3 separate experiments using 3–4 mice per strain. PK resistance was calculated by measuring the signal relative to the undigested sample.

### PrP^Sc^ solubility assay

Brain homogenates were solubilized in 10% sarcosyl in PBS and digested with 50 μg/mL of proteinase K (final concentration) at 37 °C for 30 min. Protease inhibitors were added (Complete TM™), and samples were layered over 15% Optiprep™ and centrifuged at 18,000 g for 30 min at 4 °C. Supernatants were removed and pellets were resuspended in PBS in a volume equivalent to the supernatant. Supernatant and pellet fractions were immunoblotted using anti-PrP antibody POM19. PrP signals were captured and quantified using the Fuji LAS 4000 imager and Multigauge V3.0 software. Brain samples from 3 mice were measured per strain.

### PTAA staining and analysis of frozen tissue sections

Frozen sections from mouse brain were dried for 1 hour and fixed in 100% and 70% ethanol for 10 min each. After washing with deionized water, sections were equilibrated in 100 mM sodium carbonate at pH 10.2 for 30 min. The PTAA was diluted in the sodium carbonate buffer (1 μg: 100 μl buffer) and added to the sections. The sections were incubated with PTAA for 30 min at room temperature and washed with sodium carbonate buffer. The emission spectra of PTAA bound to PrP aggregates was collected using an inverted LSM 780 confocal microscope (Carl Zeiss, Oberkochen, Germany) with excitation wavelength at 488 nm and the spectra were collected from 8 individual spots within 3–5 plaques from a minimum of two different cases of each prion-infected brain.

### Purification of PrP^Sc^ for structural studies by mass spectrometry

Samples were purified from brain homogenates as previously described^63^. Briefly, 10% brain homogenate was mixed with 5% sarcosyl (final) in TEN(D) buffer (50 mM Tris-HCl, 5 mM EDTA, 665 mM NaCl, 10% sarkosyl, 0.2 mM dithiothreitol, Complete-TM protease inhibitors (PI) (Roche), pH 8.0), incubated on ice for 1 hour, and then centrifuged at 18,000 g for 30 min at 4 °C. All but 100 μl of supernatant was removed, and the pellet was resuspended in 100 μl of residual supernatant and diluted to 1 ml with 10% sarkosyl TEN(D). Each supernatant and pellet was incubated for 30 min on ice and then centrifuged at 22,000 g for 30 min at 4 °C. Supernatants were recovered while pellets were held on ice. Supernatants were added separately into ultracentrifuge tubes with 10% sarcosyl TEN(D) buffer containing PI and centrifuged at 150,000 g for 2.5 hours at 4 °C. Supernatants were discarded while pellets were rinsed with 100 μl of 10% NaCl in TEND buffer with 1% sulfobetaine (SB 3–14) and PI and then combined and centrifuged at 225,000 g for 2 hours at 20 °C. The supernatant was discarded and pellet was washed and then resuspended in ice cold TMS buffer containing PI (10 mM Tris-HCl, 5 mM MgCl2, 100 mM NaCl, pH 7.0). Samples were incubated on ice overnight at 4 °C. Samples were then incubated with 25 units/ml endonuclease (benzonase, Sigma-Aldrich) and 50 mM MgCl_2_ for 30 min at 37 °C at 1000 rpm followed by a digestion with 10 μg/ml PK for 1 hr at 37 °C at 1000 rpm. PK digestion was stopped by incubating samples on ice with 2 mM phenylmethylsulfonyl fluoride (PMSF) for 15 min. Samples were incubated with 20 mM EDTA for 15 min at 37 °C at 1000 rpm. An equal volume of 20% NaCl was added to all tubes followed by an equal volume of 2% SB 3–14 buffer. For the sucrose gradient, a layer of 0.5 M sucrose, 100 mM NaCl, 10 mM Tris, and 0.5% SB 3–14, pH 7.4 was added to ultracentrifuge tubes. Samples were then carefully transferred and the tubes topped with TMS buffer. Samples were centrifuged at 200,000 g for 2 hours at 20 °C. The pellet was rinsed with 0.5% SB 3–14 in PBS. Pellets were resuspended in 50 μl of 0.5% SB 3–14 in PBS and stored at −80 °C.

### Backbone amide hydrogen/deuterium exchange mass spectrometry experiments (HXMS)

To initiate deuterium labeling, 10 μl aliquots of purified prions (~1–2 μg) were collected by centrifugation (21,000 g, 5 min) and suspended in 100 μl of 10 mM phosphate buffer (pH 7.3) in D_2_O. After incubation at 37 °C for 24 hours, samples were collected by centrifugation and dissociated into monomers by adding 20 μl of an ice cold exchange quench solution (100 mM phosphate, pH 2.5) containing 7 M GdnHCl and 0.1 M Tris (2-carboxyethyl) phosphine hydrochloride. After 5 minutes incubation on ice, the solution was diluted 10 times with ice cold 0.05% trifluoracetic acid and digested for 5 min using 100 μl of agarose-immobilized pepsin slurry (Thermo Scientific, Waltham, MA) as described previously^41^. The peptic fragments were collected in a C18 trap column, washed to remove salts, and separated on an UPLC BEH-C18 column (Waters, USA). To minimize back-exchange, separation was performed using a “rapid” gradient of 2–45% acetonitrile with a total elution time of 13 min, and both the trap and the analytical column were placed in a cooled chamber (~2 °C) integrated with a LEAP TriValve system (LEAP Technologies, USA). Separated peptides were analyzed by LC-MS/MS in ESI mode using an LTQ-Orbitrap XL mass spectrometer (ThermoElectron, San Jose, CA) directly coupled to the UPLC system. The mass spectrometer (externally calibrated using a Pierce ESI positive ion calibration solution) was operated in a data-dependent MS to MS/MS switching mode with the six most intense ions in each full MS scan subjected to MS/MS for further fragmentation. Full scan experiments were acquired in the m/z 300–1800 range at a resolution 60,000 (FWHM at m/z 400) and the subsequent MS/MS analysis was performed at 15,000 resolution. The following instrument parameters were used: capillary temperature, 250 °C; sheath gas, 10; auxiliary gas, 2; sweep gas, 1; spray voltage, 49 kV; tube lens, 110 V. The total scan cycle frequency was about 1 sec. The precursor ion isolation width was set at m/z ± 8.0, allowing transmission of the M and M+2 isotopic ions of the peptide for CID. The threshold intensity for the MS/MS trigger was set at 2,000 and fragmentation was carried out in the CID mode with a normalized collision energy (NCE) of 35. All data were collected in the profile mode. Chromeleon software and Xcalibur software (version 2.1, Thermo Scientific, San Jose, CA) were used for instrument control, data acquisition, and data processing. Peptide masses were calculated from the centroid of the isotopic envelope using MagTran software, and the extent of deuterium incorporation in each peptic fragment was determined from mass spectra (with a correction for back-exchange) as described previously^39^.

### Peptide mapping

Before H/D exchange experiments, pepsin digestion fragments were identified by a standard procedure involving separation on a C18 column coupled to a LTQ Orbitrap XL mass spectrometer and sequencing by tandem MS/MS using MassMatrix search engine^64^,^65^ (MassMatrix Xtreme 3.0.9.7 Alpha with 4 M spectra limit; http://magneto.case.edu/mm-cgi/home.py) against the database containing the sequence of mouse prion protein. The separation was performed using a “slow” gradient of 2–45% acetonitrile with a total elution time of 23 min.The search was performed using the “NO enzyme” search parameter. Mass tolerance of ±15 ppm and ±0.8 Daltons was used for parent and monoisotopic fragment ions, respectively. The resulting DAT files generated by MassMatrix were used as input files for peptide identification, with a constraint that only MassMatrix ion scores greater than 20 were considered. Peptide identification was further confirmed by manual inspection. Total of 99 peptides were identified (see Table S1). The peptic digest was also analyzed using FindPept^66^ on the ExPASy^67^ Proteomics server (Swiss Institute of Bioinformatics), allowing identification of longer N-terminal peptides corresponding to residues 81–132, 85–132 and 89–132 (Table S1). It should be noted that only a fraction of peptic fragments identified by peptide mapping could be separated and analyzed with good signal-to-noise ratio under the conditions of rapid UPLC gradient required for HXMS expeiments to minimize back exchange.

### Histidine hydrogen/deuterium exchange (His-HXMS) experiments

For these measurements, samples of purified PrP^Sc^ from brain (~1–2 μg) were suspended in D_2_O buffer (10 mM sodium phosphate, 10 μM EDTA, 50 μM Pefabloc, 1 ug/ml Aprotinin, pH 9.0). After incubation for 5 days at 37 °C, samples were collected by centrifugation and deglycosylated with PNGase. To obtain fragments containing single His residues, samples were then dissociated and digested with immobilized pepsin as described above for HXMS experiments, followed by digestion with silica-immobilized trypsin (2 μl; Princeton Separations, Inc., Adelphia, NJ). Finally, the peptic fragments were separated on an an UPLC BEH-C18 column (Waters, USA) and analyzed by mass spectrometry as described above for HXMS experiments. The following single-His fragments were used for His-HXMS analysis: His 95, GQGGGTHNQWNKPSKPK; His 110, HVAGAAAAGAVVGGLGG; His 139, MSRPMIHFGND; His 176, YSNQNNFVHD or YSNQNNFVHDCVN; His 186, QHTVTTTTK or ITIKQHTVT. The half-life (*t*_1/2_, days) of His exchange reaction was calculated as described previously^48^,^49^.

## Additional Information

**How to cite this article:** Aguilar-Calvo, P. *et al*. Post-translational modifications in PrP expand the conformational diversity of prions *in vivo.* *Sci. Rep.* **7**, 43295; doi: 10.1038/srep43295 (2017).

**Publisher's note:** Springer Nature remains neutral with regard to jurisdictional claims in published maps and institutional affiliations.

## Supplementary Material

Supplementary Figures and Table

## Figures and Tables

**Table 1:** Survival periods in WT and Tg(GPI-PrP) mice (days).

Strain	WT mice	Tg(GPI^−^ PrP) mice	WT mice challenged with the GPI-anchorless strain
RML	146 ± 3	142 ± 11 (5)*	147 ± 0
22 L	141 ± 2	176 ± 1 (4)	167 ± 2
mCWD	550 ± 5	160 ± 9 (4)	173 ± 4
ME7	157 ± 2	182 ± 2 (4)	230 ± 2

^*^Passage number is in parentheses.

**Table 2:** RML and GPI^-^RML prions differ in the oligomerization state and show profound differences in the disease phenotype.

Property	RML in WT mice	GPI-RML in GPI-anchorless PrP expressing mice
**Organ tropism**	Lymphoid tissue, CNS	Lymphoid tissue, CNS, adipose tissue, skeletal muscle, heart, colon (lamina propria), pancreas (islets of Langerhans), kidney
**Neuroinvasion of brain by IO, IP, IV routes**	Efficient	Poor, with 25–73% attack rate
**Neuroinvasion of brain by IN or IT routes**	Efficient	Poor, no prions in brain
**PrP**^**Sc**^ **aggregate morphology in brain**	Diffuse PrP^Sc^ surrounding astrocytes and/or neurons and in neuropil	Dense fibrillar angiocentric plaques
**PrP**^**Sc**^ **distribution in brain**	No vascular PrP^Sc^; diffuse PrP^Sc^ deposits particularly in the hippocampus, thalamus, cerebral cortex, cerebral peduncles and septum	Surrounding capillaries and widely distributed throughout the brain
**PK resistance**	Low	Higher than GPI-anchored state
**Conformational stability**	0.98+/−0.02	3.21+/−0.09 (3^rd^ psg)

IC: intracerebral; IP: intraperitoneal; IN: intranerval; IT: intratongue; IV: intravenous; IO: intraocular.

Data included are from this study and references^28^,^32^,^53^,^54^,^68^,^69^,^70^,^71^,^72^,^73^.
