# Supplementary material for: Post-translational modifications in PrP expand the conformational diversity of prions in vivo
Source: Sci Rep. 2017 Mar 8;7:43295. doi: 10.1038/srep43295 (PMC5341109; doi:10.1038/srep43295)
Supplement: Supplementary Figures and Table [file srep43295-s1.pdf]

Supplementary Information

Post-translational modifications in PrP expand the conformational diversity of prions *in vivo*

Patricia Aguilar-Calvo[1], Xiangzhu Xiao[2], Cyrus Bett[1^], Hasier Eraña[3], Katrin Soldau[1], Joaquin Castilla[3,4], Peter R. Nilsson[5], Witold K. Surewicz[2], and Christina J. Sigurdson[1,6]*

[1]Departments of Pathology and Medicine, UC San Diego, La Jolla, CA 92093-0612, USA
[2]Department of Physiology and Biophysics, Case Western Reserve University, Cleveland, OH 44116, USA
[3]CIC bioGUNE, Parque Tecnológico de Bizkaia, Ed. 800, Derio 48160, Spain
[4]IKERBASQUE, Basque Foundation for Science, 48013 Bilbao, Spain
[5]Department of Physics, Chemistry and Biology, Linköping University, Linköping 581 83, Sweden
[6]Department of Pathology, Immunology, and Microbiology, UC Davis, Davis, CA 95616, USA

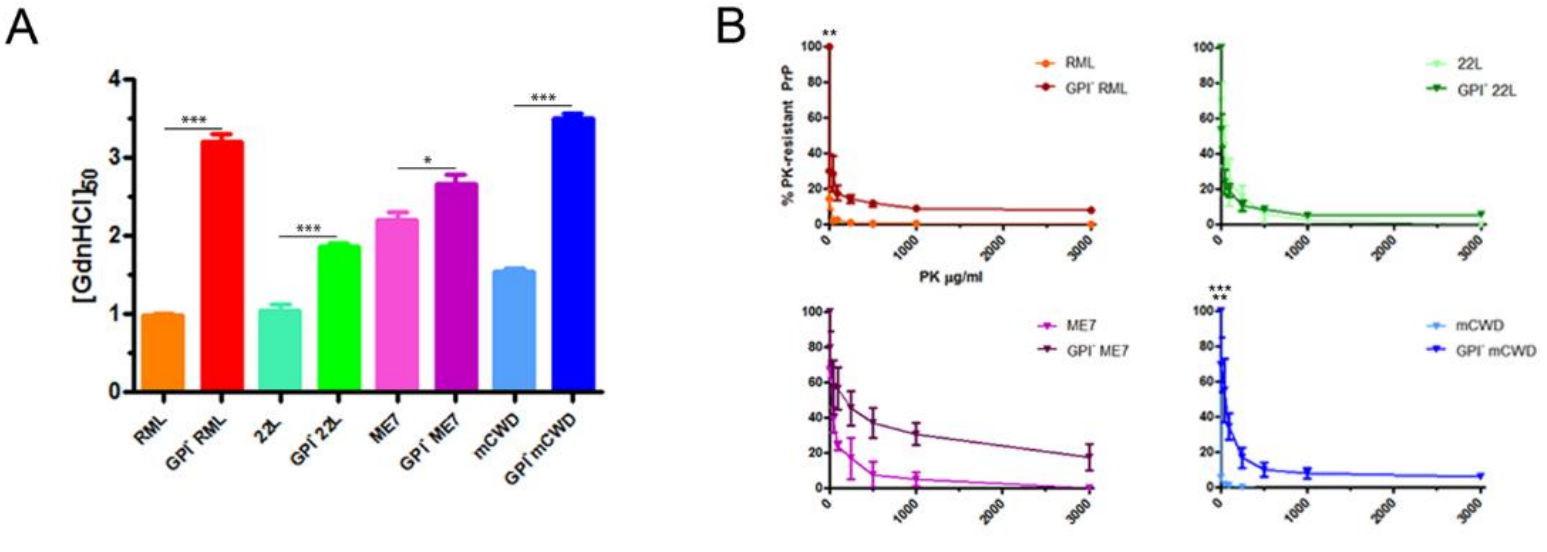

Supplementary Figure S1. Conformational stability and PK-resistance of GPI-anchorless prions in comparison with their original strains. (A) Post GPI^{-} RML, $^{-}$22L, $^{-}$mCWD and $^{-}$ME7 prions treated with different concentrations of GdnHCl followed by PK digestion are significantly more stable than their respective original GPI-anchored strains in WT mice (RML, 22L, ME7) or *tga*20 mice (mCWD) (2-tailed, unpaired Student's *t* test). RML prions showed the most dramatic increase in stability in the anchorless state (328% increase) (RML : 0.98 versus GPI^{-} RML : 3.21 ; p=0.001, Student's unpaired, two-tailed t-test). In contrast, ME7 prions showed only a slight increase (21%) in the anchorless state (ME7: 2.20 versus GPI^{-} ME7: 2.67; p=0.001, Student's unpaired, two-tailed t-test). (B) PK-resistance of GPI-anchored and anchorless RML, 22L, mCWD and mCWD. GPI^{-} RML and $^{-}$mCWD showed increased resistance to PK digestion than their anchored strains (two-way ANOVA followed by Bonferroni post-tests of GPI-anchored and anchorless prions revealed significant differences in RML at 50 µg/ml PK**, and mCWD at 10, 50, and 100 µg/ml PK***). *$P < 0.05$; **$P < 0.01$; ***$P < 0.001$.

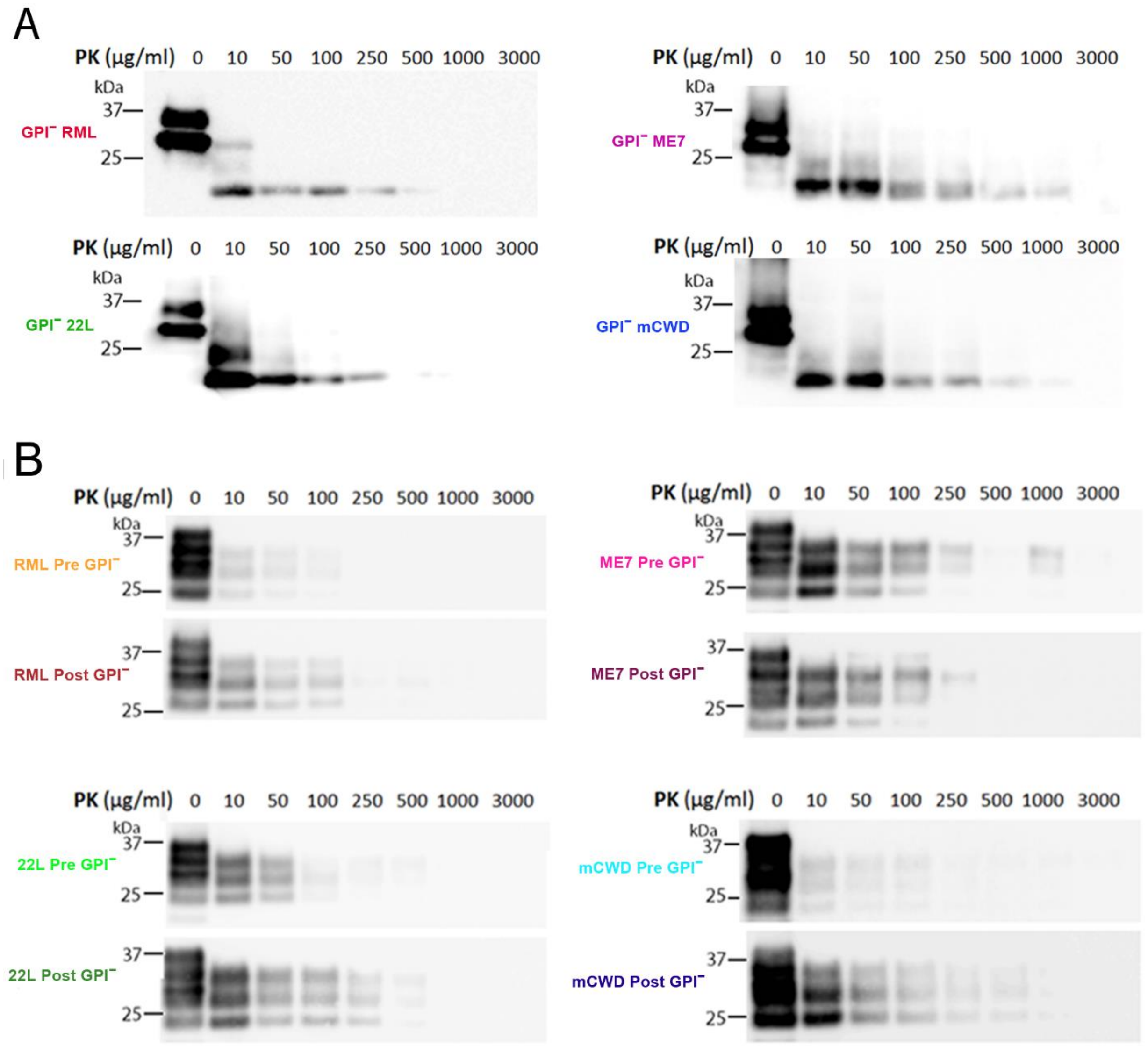

Supplementary Figure S2. Proteinase K resistance of GPI-anchorless and corresponding anchored prion strains. (A) Western blots show the PK-resistance profile of GPI⁻ RML, ⁻22L, ⁻mCWD and ⁻ME7 prions assessed after 2 hours of PK digestion at 37 °C. (B) Western blots of the PK-resistance profile of Pre and Post GPI⁻ strains.

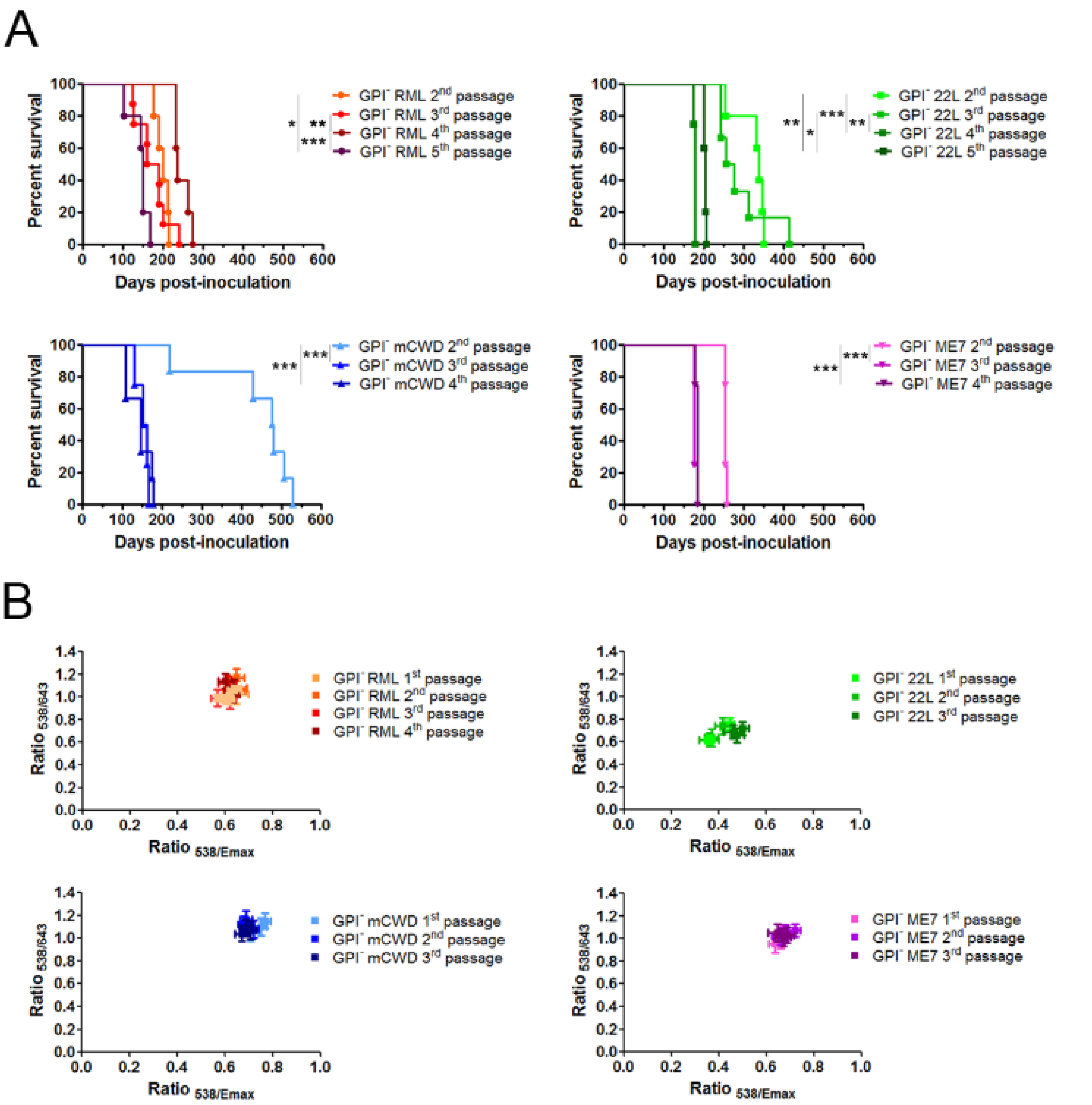

Supplementary Figure S3. Survival times and PTAA emission spectra of prion strains serially passaged in Tg(GPI-PrP) mice. (A) Survival curves of GPI^{-} RML, $^{-}$22L, $^{-}$mCWD and $^{-}$ME7 strains show a progressively decrease with passage in the GPI-anchorless mice. The longer incubation period for the fourth passage of RML may due to titer differences among individual mice used for the inoculations. (B) PTAA bound to GPI^{-} RML, $^{-}$22L, $^{-}$mCWD and $^{-}$ME7 PrP^{Sc} plaques through serial passages in GPI-anchorless mice. No significant differences in the ratios of emitted light intensity at ratios of 538 nm / 643 nm and 538 nm / emission maximum were observed between 1st, 2nd, 3rd or 4th serial passages of GPI^{-} RML, $^{-}$22L, $^{-}$mCWD and $^{-}$ME7 strains in the GPI-anchorless mice. For survival times, n= 4-6 mice per group except 4th passage 22L (n=3) and 2nd passage mCWD (n=15). One-way ANOVA followed by Tukey's multiple comparison test for survival times, *P < 0.05; **P < 0.01; ***P < 0.001.

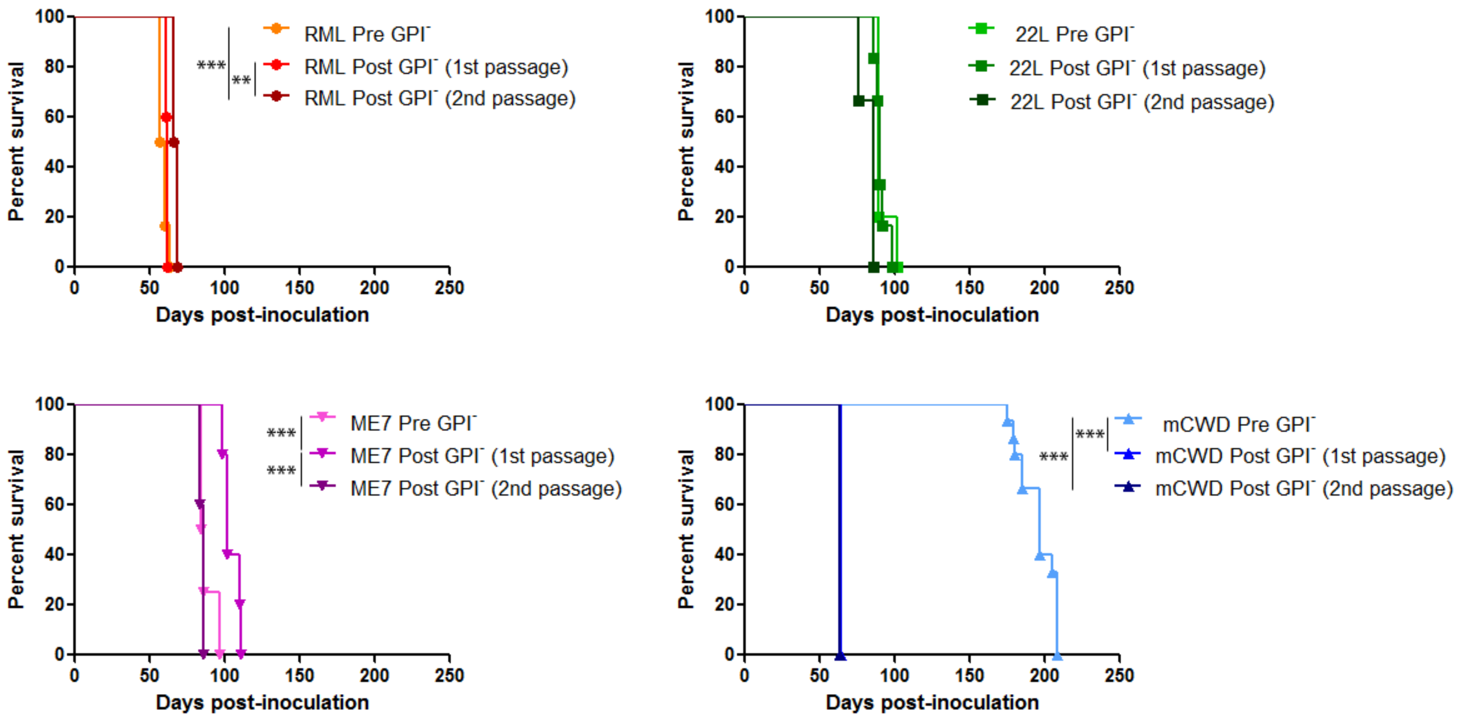

Supplementary Figure S4. Survival curves of *tga*20 mice inoculated with GPI-anchored (Pre GPI$^-$) and anchorless strains (Post GPI$^-$). Post GPI$^-$ RML, -22L and -ME7 retained the same survival times of the original anchored strains in *tga*20 mice (first and second passage), while Post GPI$^-$ mCWD showed a four times shorter survival time than anchored mCWD. Note, the survival times for first and second passages overlap (64 ± 0 versus 63 ± 0 days post inoculation, respectively). N=4-5 mice per group. One-way ANOVA followed by Tukey's multiple comparison test for survival times, $^{**}P < 0.01$; $^{***}P < 0.001$.

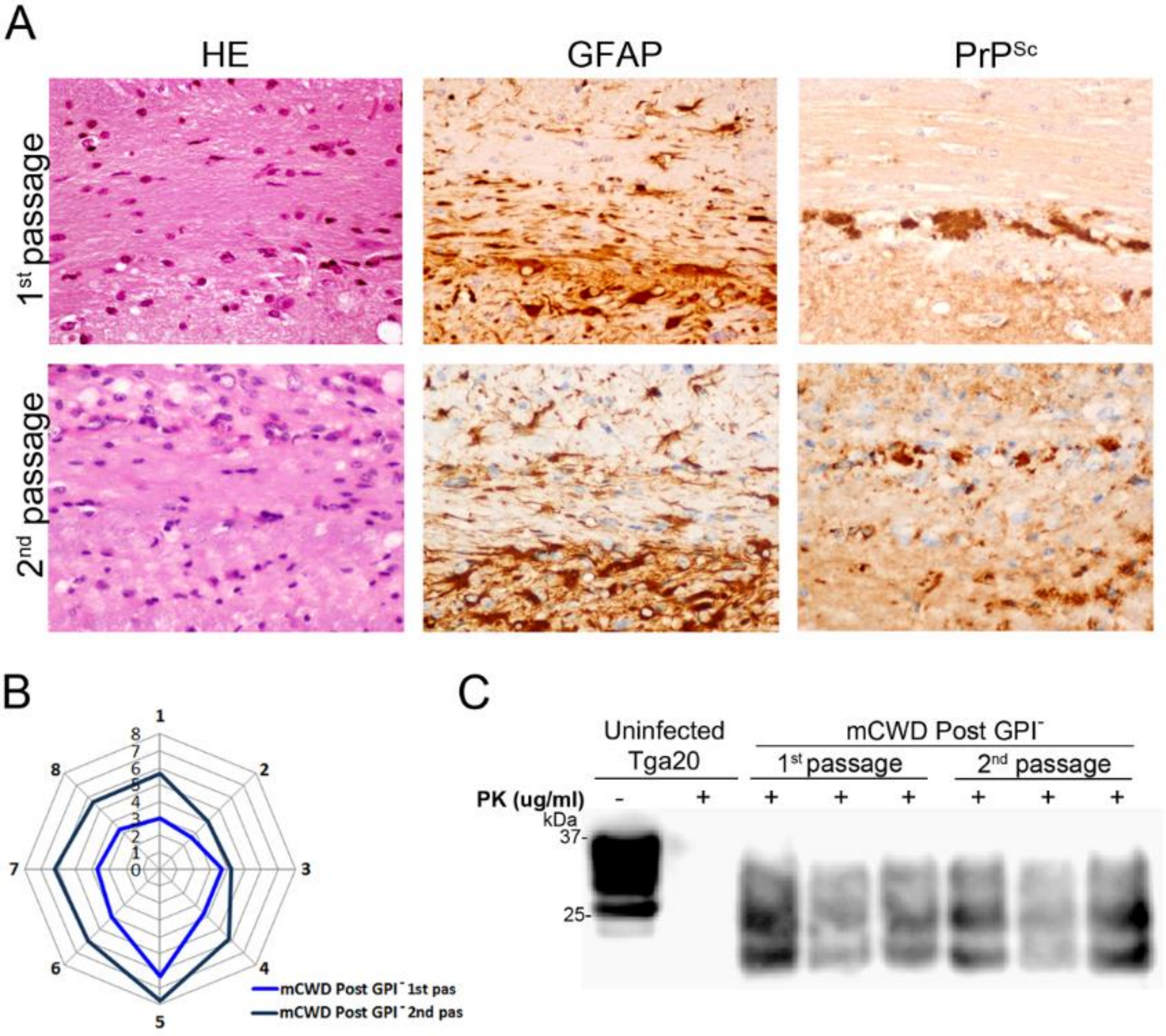

Supplementary Figure S5. Histologic properties of Post GPI$^-$ mCWD strain at first and second passage in Tga20 mice. (A) PrP^{Sc} aggregates in the brain (corpus callosum) of GPI$^-$ mCWD infected *tga*20 mice at first passage forms small dense aggregates accompanied by mild vacuolation and gliosis, yet shows more severe gliosis upon second passage. (B) A lesion profile reveals PrP^{Sc} aggregates primarily in the hippocampus in the first passage mice, while PrP^{Sc} accumulation is more severe by the second passage. (C) The electrophoretic mobility of GPI$^-$ mCWD PrP^{Sc} from the first and second passage in *tga*20 mice was similar.
